# Petersen’s space hernia as a complication of gastric bypass surgery: a case report

**DOI:** 10.1093/jscr/rjae504

**Published:** 2025-02-14

**Authors:** Mauricio Fabian Palacios, Alex Paul Guachilema Ribadeneira, Julio Yepez, Andrea Lisintuña Cisneros, Sandra Morocho Imbacuan, Sergio Verboonen

**Affiliations:** Department of General Surgery, Hospital Metropolitano, Quito 170521, Ecuador; Department of General Surgery, Hospital Metropolitano, Quito 170521, Ecuador; Department of Internal Medicine, Hospital Metropolitano, Quito 170521, Ecuador; Department of General Surgery, Hospital Metropolitano, Quito 170521, Ecuador; Department of Anesthesiology, Hospital General Enrique Garces, Quito 170608, Ecuador; Department of Bariatric Surgery, Obesity Goodbye Center, Tijana 22000, Mexico

**Keywords:** internal hernia, Petersen’s hernia, bariatric surgery, gastric bypass, laparoscopy, intestinal obstruction

## Abstract

Internal hernia is the most common complication of Roux-en-Y gastric bypass. Although its incidence rate is low, hernias are becoming more common in patients undergoing bariatric surgery, with Petersen’s hernia being one of the most frequent. The symptoms of internal hernia are variable, and the sensitivity of imaging methods is limited, resulting in a high rate of misdiagnosis of internal hernia by computed tomography. Surgery continues to be the first treatment option in patients presenting with clinical symptoms of obstruction after undergoing Roux-en-Y gastric bypass. Here, we present a case of intestinal obstruction secondary to Petersen’s hernia after Roux-en-Y gastric bypass in the context of bariatric surgery.

## Introduction

Bariatric surgery is currently considered the most effective treatment method for long-term weight loss, and a common surgical technique is Roux-en-Y gastric bypass; however, internal hernia is the most frequent cause of intestinal obstruction after gastric bypass in these patients [1].

Petersen’s hernia was first described by Walther Petersen, a German surgeon, and only 178 incidents were reported until 1974; however, the use of laparoscopic gastric bypass for the treatment of obesity has increased exponentially, resulting in an increased incidence of Petersen’s hernia in recent years [2]. Hernias occur in 0.5%–11% of patients after surgery, and internal hernias resulting from laparoscopic bariatric surgery have a higher incidence than those after open surgery. Clinical data and studies indicate that internal hernias can be a recurrent problem with a recurrence rate as high as 19% [3].

Although symptoms can occur at any time after surgical intervention, the incidence of small bowel obstruction is highest between 1 and 2 years after surgery [4, 5].

Hernias are the main cause of abdominal pain in the late postoperative period and they usually present with diffuse abdominal pain [6].

Computed tomography (CT) plays an important diagnostic role, and although CT findings are often inconclusive, the mesenteric whirl sign is one of the most effective indicators of internal hernia after bypass surgery [2].

## Clinical case presentation

**Figure 1:**
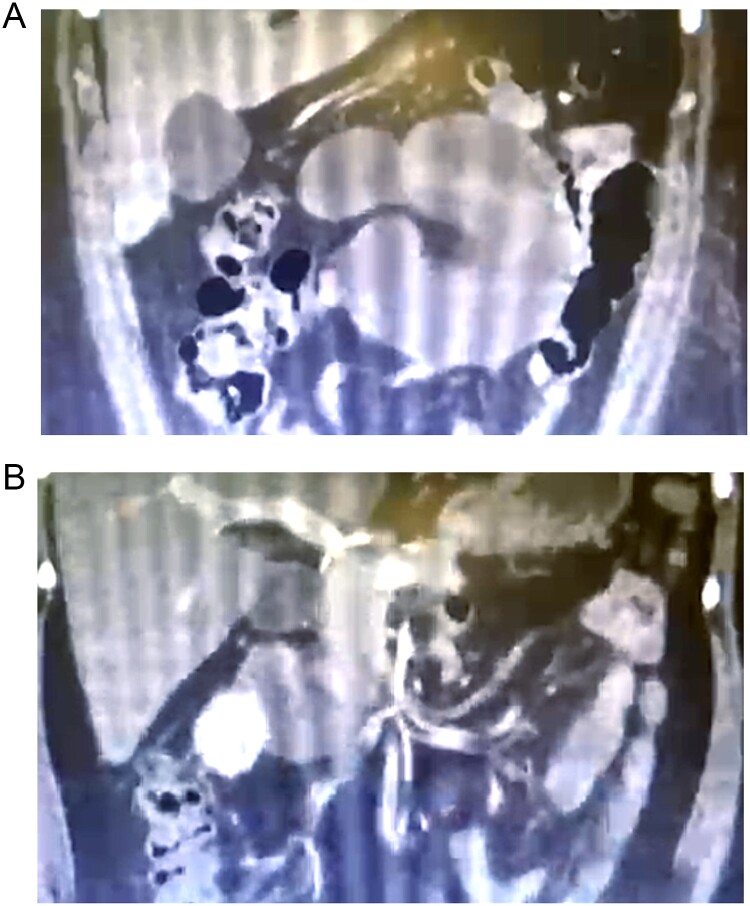
(A) Abdominal tomography showing herniation of the intestinal loops toward the space posterior to the gastrojejunostomy; (B) dilated and congested intestinal loops associated with torsion and congestion of mesenteric vessels.

A 36 year-old woman with a history of obesity, gastroesophageal reflux, and hiatal hernia who underwent laparoscopic Roux-en-Y gastric bypass with hiatoplasty was discharged at 24 hours after surgery without complications. Three days later, the patient presented to the emergency room with complaint of sudden high-intensity postprandial abdominal pain of a colicky type, localized in the epigastrium and radiating to the hypogastrium, accompanied by abdominal distension and lack of flatus. Physical examination revealed a dry oral mucosa, pale skin, decreased vesicular breath sounds in both lungs, tense abdomen in the superior hemiabdomen, and diffuse tenderness on palpation with absent hydro-aerial sounds. Contrast CT of the abdomen and pelvis revealed dilated intestinal loops that were congested and associated with torsion and congestion of the mesenteric vessels, herniation of intestinal loops toward the space posterior to the gastrojejunostomy, and a moderate amount of free fluid in the pelvic cavity. There was no evidence of free air (Fig. 1A and B). The patient was referred for surgical revision.

**Figure 2:**
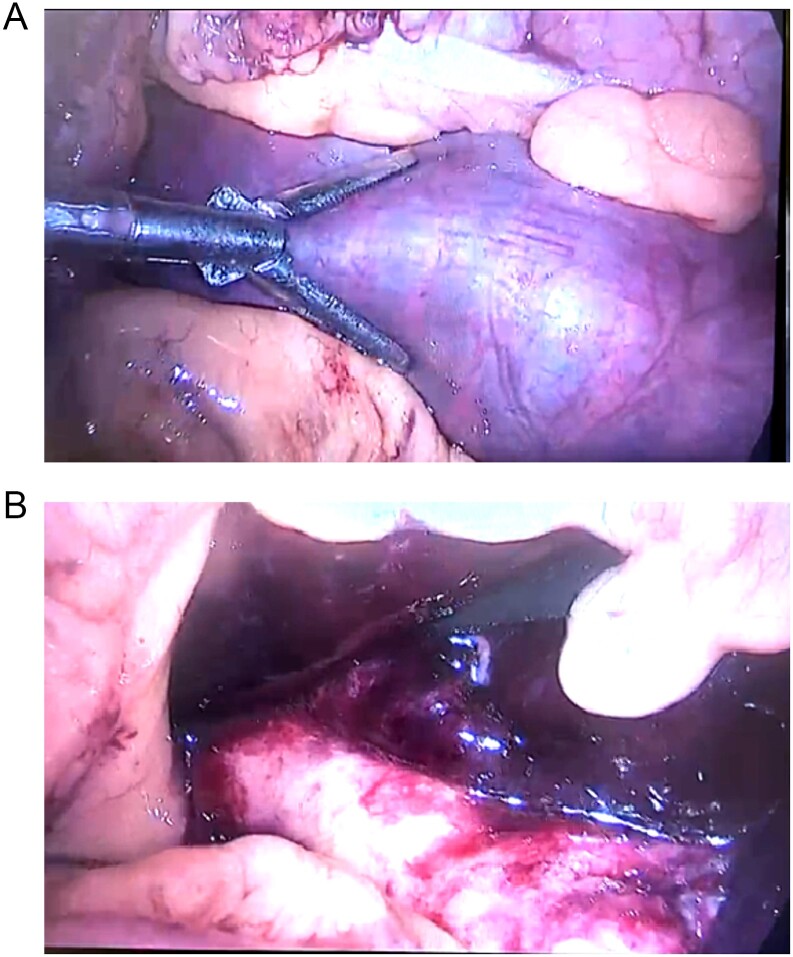
(A) Intestinal loops in Petersen’s space; (B) a 70 cm segment of necrotic jejunum starting at 15 cm from the angle of Treitz.

**Figure 3:**
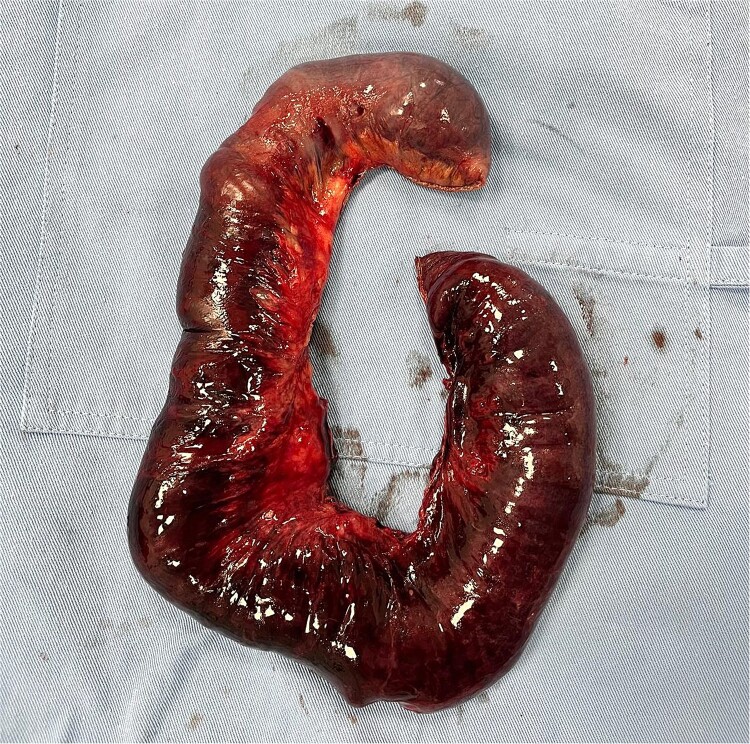
Surgical sample for pathology.

Revision surgery was performed, and the findings included herniation of intestinal loops through Petersen’s space, a segment of necrotic jejunum measuring 70 cm starting at 15 cm from the angle of Treitz (Fig. 2A and B), intact entero-enteric anastomosis, and intact alimentary and common limbs. Bowel resection and entero-enteric anastomosis were performed with a 45 mm linear auto suture (Fig. 3). A drain tube was inserted toward the anastomosis, and oral feeding was initiated in the immediate postoperative period, which was well tolerated. The patient was discharged on postoperative Day 3 in good condition.

Histopathological report: ischemic infarction secondary to intestinal obstruction.

## Discussion

Intestinal obstruction caused by an internal hernia has been documented as a possible complication after Roux-en-Y gastric bypass. This complication is due to prolapse of intestinal loops through the mesenteric defect generated by the Y-shaped reconstruction performed during gastric bypass. Two defects can be generated as a consequence of antecolic reconstruction: the first one is the defect generated by the mesenteric interruption in the location of the jejuno-jejunal anastomosis, and the second one is known as Petersen’s space, which is circumscribed by the alimentary loop and the transverse mesocolon [1]. An incidence rate of 0.5%–11% has been reported in the literature as well as a cumulative incidence of 4%–7% without closure of any defects [2].

Once a connection between the stomach and the small bowel is established through any type of gastrojejunal anastomosis, a space forms behind the limbs of the small intestine, which can potentially lead to an internal hernia. Another factor that contributes to the development of an internal hernia is that as patients lose weight, the anatomy of the intestine undergoes changes, and any existing mesenteric defect can be exacerbated or new defects can emerge [1].

The clinical presentation is nonspecific, which is associated with considerable morbidity, and it can vary from slight abdominal discomfort to intense pain. The severity of symptoms depends on the duration of the hernia and the presence of strangulation or incarceration [1, 2, 6].

The radiological diagnosis of internal hernia is achieved using CT. The characteristic radiological signs are as follows: (i) mesenteric swirl of vessels and fat at the root of the mesentery; (ii) mesenteric vessel engorgement; (iii) small bowel passing behind the superior mesenteric artery; and (iv) right-sided location of the jejuno-jejunal anastomosis at the right of the alimentary canal and anterior displacement toward the right side of the Treitz ligament with sensitivity and specificity of 94%–100% and 90%–95%, respectively [1, 2].

Negative CT findings are not sufficient to rule out the possibility of an internal hernia, and surgical exploration remains the gold standard in cases of clinical suspicion [1, 2, 4].

There are many methods available for preventing complications during or after Roux-en-Y antecolic reconstruction. One popular method among surgeons is the closure of defects using non-absorbable suture to facilitate the formation of adhesions necessary to ensure mesenteric closure when the patient loses weight, thereby decreasing the possibility of a hernia [2, 4].

In the present case, a complication of laparoscopic gastric bypass resulted in the development of Petersen’s hernia, leading to abdominal obstruction with strangulation and necrosis of the small intestine in a potentially fatal complication. This case was resolved laparoscopically with excellent recovery.
